# Delinking Investment in Antibiotic Research and Development from Sales Revenues: The Challenges of Transforming a Promising Idea into Reality

**DOI:** 10.1371/journal.pmed.1002043

**Published:** 2016-06-14

**Authors:** Kevin Outterson, Unni Gopinathan, Charles Clift, Anthony D. So, Chantal M. Morel, John-Arne Røttingen

**Affiliations:** 1 Boston University School of Law, Boston, Massachusetts, United States of America; 2 Chatham House Centre for Global Health Security, London, United Kingdom; 3 DRIVE-AB, Innovative Medicines Initiative, Geneva, Switzerland; 4 Oslo University Hospital, Oslo, Norway; 5 Norwegian Institute of Public Health, Oslo, Norway; 6 Johns Hopkins Bloomberg School of Public Health, Baltimore, Maryland, United States of America; 7 ReAct - Action on Antibiotic Resistance, Uppsala, Sweden; 8 London School of Economics, London, United Kingdom; 9 University of Oslo, Oslo, Norway; 10 Harvard School of Public Health, Boston, Massachusetts, United States of America

## Abstract

Kevin Outterson and colleagues outline a model to address access, conservation, and innovation of antibiotics.

**Summary Points**

1. The current business model for antibiotics is plagued by market failures and perverse incentives that both work against conservation efforts and provide insufficient rewards to drive the development of much-needed new treatments for resistant infection.
2. Many new incentive mechanisms have been proposed to realign incentives and support innovation and conservation over the long term. The most promising of these are based on the idea of delinking rewards from sales volume of the antibiotic—the notion of “delinkage.”
3. Some critical design issues for delinkage remain, such as how to secure access to badly needed new products when resistance renders existing treatments ineffective, an increasingly urgent global problem. The issue of global access to antibiotics is not sufficiently addressed de facto by a delinked mechanism, and, as such, it must be addressed explicitly through specific design features of new mechanisms, including defining the eligibility criteria for delinkage rewards and appropriate management of intellectual property.
4. The idea of establishing a new business model to drive antibiotic development and improve conservation currently has the world’s attention. We must now work quickly to examine the remaining design questions to address this major public health concern for the longer term.

## Introduction

Senior political leaders are now aware of the threat to human health and, indeed, the global economic toll posed by the growing level of antibiotic resistance. An Executive Order issued in 2015 by US President Barack Obama addressed antibiotic resistance [1], and a US national action plan has been developed [2]. It is one of German Chancellor Angela Merkel’s three health priorities for the G7 during her presidency. In May 2015, the World Health Organization (WHO) member states agreed on a global action plan on antimicrobial resistance [3]. The Review on Antimicrobial Resistance (AMR Review), commissioned by UK Prime Minister David Cameron, has estimated that antimicrobial resistance could result in 300 million premature deaths over the next 35 years and result in a global gross domestic product (GDP) loss of 2%–3.5% by 2050 [4].

Most of the commonly prescribed classes of antibiotics were discovered before the 1960s, with only a few novel classes being discovered and approved for clinical use in recent decades [5–7]. The innovation system for discovering new drugs has not been able to keep up with increasing levels of resistance, with WHO warning that “without urgent, coordinated action by many stakeholders, the world is headed for a post-antibiotic era” [8].

A number of different proposals and research and development (R&D) models for stimulating antibiotic innovation have been proposed [9,10], each differing with respect to characteristics such as the type and timing of innovation incentives and the degree of governmental public control of the market. For antibiotics, business models based on sales volumes tend to promote overuse and thereby resistance. Here we build on a recently published Chatham House report [11] focused on how policymakers could move forward in designing delinked mechanisms separating the return on investment in R&D from antibiotic sales volume and revenues. We describe three key issues, access, conservation, and innovation, and address how they could work within a delinked model for developing future antibiotics.

### Who Should Coordinate the Delinked Mechanism and How Will It Be Funded?

A new antibiotic business model must be globally coordinated, as national or regional approaches to addressing the problem will not alone ensure that benefits of antibiotic innovation are equitably shared, nor will they be sufficient to effectively address the cross-border consequences of antibiotic overconsumption. Different options exist. Coordination could be through a body hosted by WHO, in collaboration with relevant intergovernmental agencies, or be set up more independently. Stronger legal and financial commitment mechanisms could be in the form of a treaty or a WHO regulation that covers access, conservation, and innovation [12].

How more funding can be raised efficiently and equitably for unmet R&D needs has been a longstanding and largely unresolved issue in global health. For example, the Consultative Expert Working Group on Research and Development established by WHO recommended governments commit at least 0.01% of GDP for health needs in low- and middle-income countries (LMICs) [13], a target that has yet to be met by most WHO member states.

For financing antibiotic innovation through global resources, a number of incremental steps are needed. First, the global resource commitments required to tackle the challenge of antimicrobial resistance, including the cost of R&D incentives, need to be estimated. Second, that funding should be spent in line with agreed global priorities. Even if most of this funding will be under the control of national governments, a substantial proportion should be allocated to a pooled funding mechanism coordinated globally, given that parts of it will need to cover delinkage rewards. It should be noted that complete financial participation from a large number of countries may not be necessary to initiate a new business model. For example, a contribution of 0.01% of GDP from the Organization for Economic Cooperation and Development (OECD) countries alone could yield between US$4 billion and US$5 billion annually. Other members of the G20 and BRICS (Brazil, Russia, India, China, and South Africa) might also participate, as these countries constitute large antibiotic markets [14]. All countries, including LMICs initially unable to contribute financially, could make non-financial commitments to sustain antimicrobial effectiveness, such as the implementation of conservation and surveillance measures, in line with WHO’s global plan of action on antimicrobial resistance.

### Where and How Can R&D Incentives Be Most Effectively Applied in the Lifecycle?

A key question is how to attract pharmaceutical innovators, both small- and medium-sized enterprises and larger multinational companies, to increase investments in R&D for antibiotics. Drug companies want their new products to be sold, but for antibiotics, for excellent public health reasons, we want the newest antibiotics to be used sparingly. This dynamic uniquely disfavors investments in antibiotic R&D [15]. New antibiotics are also unlikely to be demonstrably superior to existing drugs, many currently available in cheaper generic forms, in part due to lack of superiority data resulting from the widespread use of non-inferiority trials.

Physicians and health systems will become increasingly cautious about prescribing new antibiotics, except for the still relatively rare case in which existing antibiotics are ineffective. For example, novel antibiotics effective against Carbapenem-resistant Enterobacteriaceae (CRE) should, for sound clinical reasons, be reserved for patients in whom the pathogen and its resistance profile are known, which is usually in a hospital setting. These antibiotics will therefore not be available for widespread use in primary care, where over 80% of antibiotics are prescribed [17]. This restricts the potential sales and use of a novel antibiotic during its market lifecycle. Having done the calculations, the potential innovator will therefore not expect to generate the revenues necessary to justify any major investment in antibiotic R&D.

**Fig 1:**
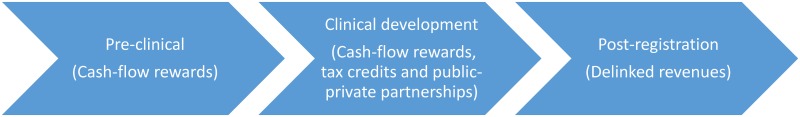
R&D incentives over the antibiotic lifecycle.

In order to increase the rewards for investing in R&D, increased incentives for R&D will be needed at three different entry points of the antibiotic lifecycle (Fig 1). At the preclinical stage, increasing public funding of antibiotic research will generate scientific advances in university laboratories and other research institutions, filling the early pipeline with potential therapeutic options. During the clinical development stage, there should be public sector support in the form of tax credits, milestone prizes or grants, and enhanced support and coordination of clinical trial infrastructure. Public–private partnerships (PPPs) in which public bodies and innovators collaboratively move drugs through the three phases of clinical trials enable each to bring their comparative advantages to the partnership.

The third entry point for incentives is after marketing approval and registration. It is at this stage that incentives are implemented to break the link whereby R&D is principally financed from revenue streams stemming from sales volume. Instead, companies producing a truly novel antibiotic should receive rewards after receiving marketing approval, linked not to sales but to R&D costs invested or potential therapeutic value. The magnitude of these delinkage rewards should be adjusted for net public investment across the R&D lifecycle. One option is to pay the innovator a significant one-time payment, adjusted to R&D costs and potential clinical value, shortly following marketing approval. This option may look attractive due to its simplicity; however, evidence of novelty and clinical value is likely to be insufficient upon marketing approval of a new antibiotic. Public funders may therefore risk rewarding innovators for drugs that over time prove unable to meet unmet clinical needs. An alternative is therefore to implement a staged approach, in which a minimum base reward covers R&D expenses and the cost of building a supply chain, and subsequent annual payments may increase depending on data regarding clinical effectiveness, resistance profiles, and other data indicating the social value of the new drug [16].

### How Can We Optimally Address Clinical Need, Global Access, and Conservation Within the Delinked Incentive Framework?

At the early preclinical stage, broad qualification standards should be applied when making grants to fund basic scientific work in antibiotic research. At the clinical and post-registration stage (after Food and Drug Administration [FDA] or European Medicines Agency [EMA] approval), incentive levels should be guided by a periodically updated global threat and needs assessment, and larger incentives should only apply to drug candidates with the potential to address significant unmet medical needs resulting from drug-resistant bacteria. Within these broad lines, the choice of target pathogen(s) has clear implications for geographic usefulness and global access. Ideally, incentives should be directed toward the development of new antibiotics intended for last-resort use as well as toward new agents with immediate therapeutic value due to high levels of resistance.

As an example, we may face, due to varying levels of antibiotic resistance across geographic regions, future scenarios in which a novel antibiotic is reserved as a last resort in high-income settings, while being required for immediate clinical use in financially impaired markets in LMICs. It is therefore important that delinkage, in addition to being an innovation incentive, also address global access. Delinkage was introduced in the global health community as an incentive mechanism in which markets and market-shaping mechanisms were insufficient or not working as signals for innovation investments. By more directly rewarding innovation, it also in effect became an access-related approach, since licensing arrangements potentially could secure competition in manufacturing, thereby allowing for generic level prices [18]. Full delinkage would divorce the incentive to recoup R&D through volume-based sales across all markets. A variation on this approach, partial delinkage, maintains higher pricing in higher-income countries. But this has the potential of exacerbating the misalignment of economic incentives, motivating companies to concentrate their efforts to reach higher-paying markets, possibly to the detriment of needed access in less-well-resourced markets.

To enable global access, a delinked incentive framework must also address the management of intellectual property (IP). Intellectual property has historically been a contentious issue with respect to global access, with the most prominent example being civil society and governments in LMICs clashing with multinational pharmaceutical companies over IP policies that restricted access to antiretroviral treatment for HIV/AIDS [19]. Struggles with IP need not be the case for antibiotics, as long as appropriate responsibilities are allocated between governments and innovators when negotiating the terms of delinkage rewards (Table 1). One alternative is that the innovator maintains ownership of IP, but, in return for delinkage rewards, makes antibiotics available to a global coordinating body and supplies markets at a price based on production costs (price being independent of the reward itself). In case the innovator wants to concentrate supply to specific geographic markets, negotiations should ideally result in the innovator foregoing a share of delinkage rewards corresponding to uncovered markets. A global coordinating body could then use these shares to incentivize and reimburse generic companies supplying markets not served by the innovator. Another option is a full patent buyout model. In this case, a global coordinating body could take responsibility for global distribution arrangements, including manufacturing, sales, global regulatory approvals, and post-marketing surveillance. A more likely scenario is that the global coordinating body decides to negotiate licensing agreements with companies willing to manufacture and supply the drug. These would, in return, receive delinkage rewards reflecting the geographic scope of territories covered.

**Table 1:** Rights and responsibilities for the patent holder and a global coordinating body (GCB) in three IP holding scenarios^1^.

IP scenario	IP rights	Antibiotic price set by	Antibiotic sales revenues accrue to	Manufacturing arranged by	Marketing authorization, post-approval studies, etc. by
Marginal cost procurement contract	Owned by innovator	At company cost to global coordinating body (GCB); set by GCB downstream	GCB	Innovator	Innovator
Partial buy-out	Licensed to GCB	GCB	GCB	Innovator (or third parties for geographic areas not covered by the innovator)	Innovator (or third parties for geographic areas not covered by the innovator)
Full buy-out	Owned by GCB	GCB	GCB	GCB, through licensing or contracts with third parties	GCB, through licensing or contracts with third parties

^1^All these options assume full delinkage. All options could potentially implement a “top-up” system requiring high-income countries to pay a higher price than the completive price close to marginal cost.

Delinkage should also have an inherent rational use component in that it ideally, in return for rewards, should commit the innovator to not overmarket and oversell the drug. The antibiotic can be used solely based on clinical needs rather than the need to achieve profitable sales targets.

Finally, antibiotic delinkage should commit innovators to appropriate sharing of information on effectiveness, distribution, sales, and other data about the product, which should be made distinct from marketing practices to the extent possible. The commitments to information sharing should be incorporated into the conditions for reward eligibility, and these should be agreed to with the innovator also in the case of partial delinkage models. Overall, a delinked incentive framework should present policymakers with options to enable global access while maintaining reasonable control over antibiotic sales and distribution.

## Transforming a Promising Idea into Reality

The delinkage principle applied to antibiotics is a powerful one and has attracted attention not just from academics and NGOs but also from key figures in industry [20], high-level policy circles in Europe and the United States, and the OECD [21]. The UK’s AMR Review, led by Lord Jim O’Neill, proposed that “a successful intervention must partially or fully ‘de-link’ profit from sales” [7]. With this growing focus on antibiotic delinkage, we see the need for a global conversation that applies delinkage principles to address access, conservation, and innovation of antibiotics in concert and not in isolation [12,22]. A global framework must, irrespective of the specific policy option chosen to achieve global collective action [23], mobilize both the sufficient level of commitment between states to address existing gaps in international collaboration and the trust in that doing so will accrue benefits immediately and over the long term [24]. Global political commitment is needed now to transform antibiotic delinkage from a promising idea into reality.
